# Supplementary figures and images for: Genome-Wide Analysis of Lipoxygenase (LOX) Genes in Angiosperms
Source: Plants (Basel). 2023 Jan 14;12(2):398. doi: 10.3390/plants12020398 (PMC9867167; doi:10.3390/plants12020398)

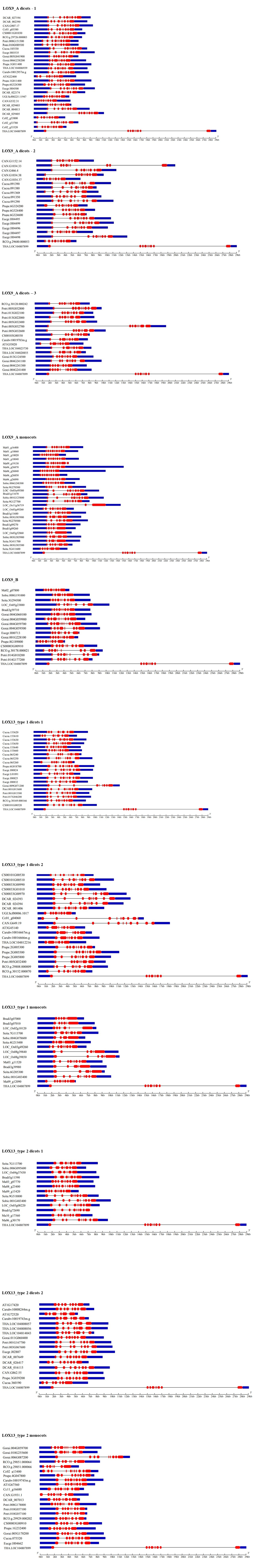

Supplement: Supplementary file 1 [file plants-12-00398-s001.zip › Supplemental Figure S1.tif]

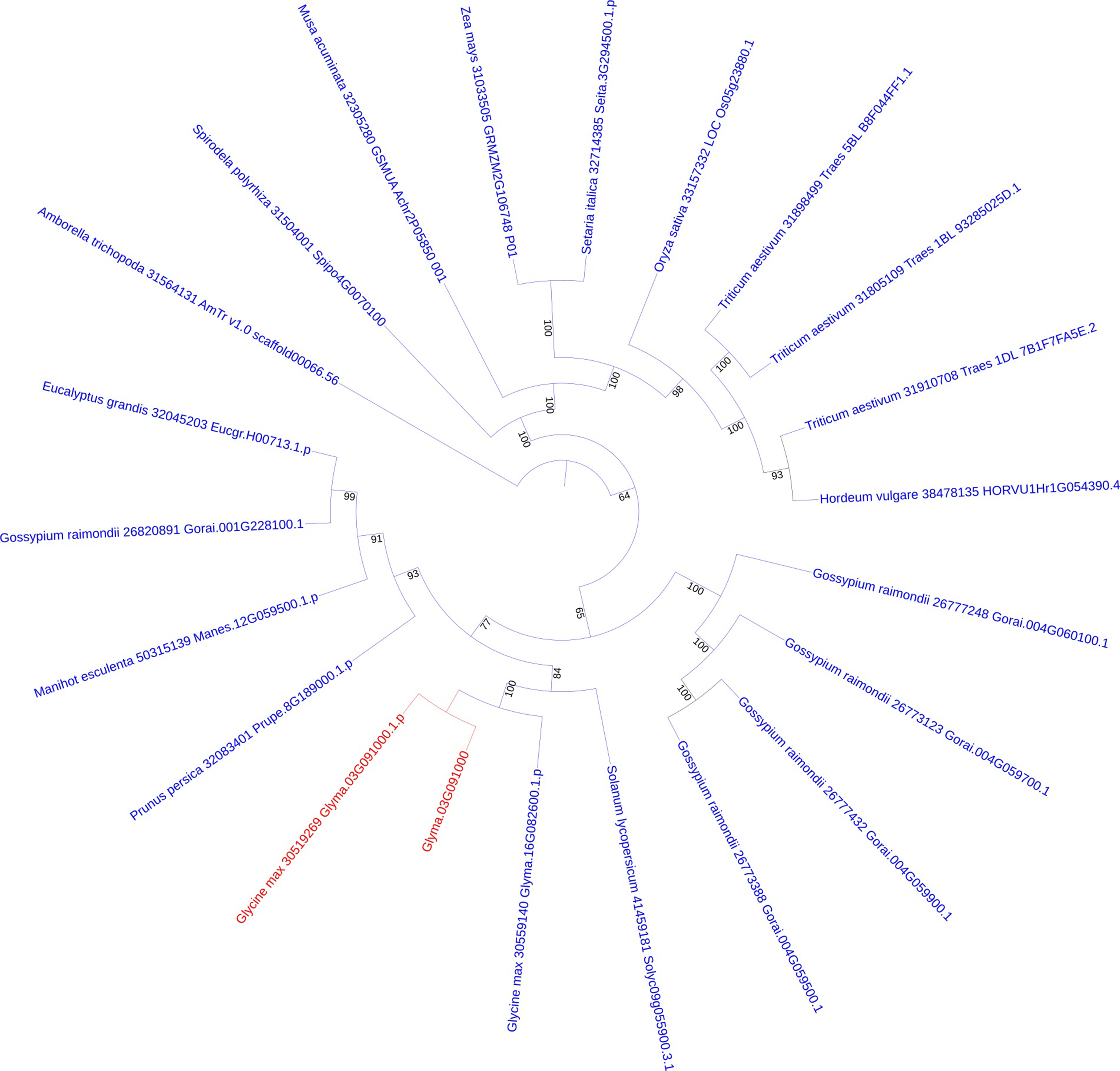

Supplement: Supplementary file 1 [file plants-12-00398-s001.zip › Supplemental Figure S2.tif]
